# Twelve Years of the Italian Program to Enhance Relational and Communication Skills (PERCS)

**DOI:** 10.3390/ijerph18020439

**Published:** 2021-01-08

**Authors:** Lidia Borghi, Elaine C. Meyer, Elena Vegni, Roberta Oteri, Paolo Almagioni, Giulia Lamiani

**Affiliations:** 1 Department of Health Sciences, University of Milan, 20142 Milan, Italy; elena.vegni@unimi.it (E.V.); giulia.lamiani@unimi.it (G.L.); 2 Boston Children’s Hospital, Boston, MA 02115, USA; elainecmeyer@gmail.com; 3 Center for Bioethics, Harvard Medical School, Boston, MA 02115, USA; 4 ASST Santi Paolo and Carlo Hospitals, 20142 Milan, Italy; oteri.roberta@gmail.com; 5 Humanitas Research Hospital, 20089 Milan, Italy; paolo.almagioni@humanitas.it

**Keywords:** bad news, clinical psychology, healthcare communication, continuing medical education, difficult conversations, post-graduate training

## Abstract

To describe the experience of the Italian Program to Enhance Relations and Communication Skills (PERCS-Italy) for difficult healthcare conversations. PERCS-Italy has been offered in two different hospitals in Milan since 2008. Each workshop lasts 5 h, enrolls 10–15 interdisciplinary participants, and is organized around simulations and debriefing of two difficult conversations. Before and after the workshops, participants rate their preparation, communication, relational skills, confidence, and anxiety on 5-point Likert scales. Usefulness, quality, and recommendation of the program are also assessed. Descriptive statistics, *t*-tests, repeated-measures ANOVA, and Chi-square were performed. A total of 72 workshops have been offered, involving 830 interdisciplinary participants. Participants reported improvements in all the dimensions (*p* < 0.001) without differences across the two hospitals. Nurses and other professionals reported a greater improvement in preparation, communication skills, and confidence, compared to physicians and psychosocial professionals. Usefulness, quality, and recommendation of PERCS programs were highly rated, without differences by discipline. PERCS-Italy proved to be adaptable to different hospital settings, public and private. After the workshops, clinicians reported improvements in self-reported competencies when facing difficult conversations. PERCS-Italy’s sustainability is based on the flexible format combined with a solid learner-centered approach. Future directions include implementation of booster sessions to maintain learning and the assessment of behavioral changes.

## 1. Introduction

Conveying difficult news to patients and families is a common occurrence in clinical practice. Communicating the onset of chronic disease or death of a loved one, approaching families for organ donation, disclosing medical error, or communicating an implantation failure to an infertile couple are just some of the difficult conversations that clinicians may face in clinical practice. The manner in which these conversations are conducted can have long-lasting effects on patients and families [1]. Several studies report that communication skills and relational abilities of clinicians are associated with greater patient satisfaction and adherence [2], improved health outcomes [3], decreased bereavement burden [1], fewer malpractice claims [4], and greater positive attitude towards healthcare organizations [5].

For clinicians, engaging in difficult conversations can represent a source of stress and result in a range of emotions that can be taxing and fatiguing, such as fear, anxiety, blame, impotence, and frustration [6,7]. Conducting difficult conversations requires clinicians to have not only a solid knowledge of communication theories and skills, but also to cultivate relational capacities such as empathy, flexibility, self-reflection, and creativity that are more nuanced and challenging to learn [8,9]. Browning et al. [10] highlighted how being competent in difficult conversations is linked to developing a sense of confidence and self-reflective capacity, appreciating the contextual uniqueness of these conversations, being able to tolerate uncertainty and imperfection, being willing to share the moral burden of decisions, and integrating personal authenticity with one’s professional role. Referring to Bloom’s taxonomy, we might say that learning how to conduct difficult conversations encompasses growth across cognitive, behavioral, and affective domains. Lipkin and colleagues [11] highlight the importance of teaching knowledge, skills, and attitudes simultaneously as this integrated approach likely produces better results than teaching each in isolation. Moreover, the importance of learning and practicing difficult conversations in interdisciplinary contexts is recognized as engendering opportunities for collaborative relationships, resources, and perspective sharing [12].

Over the last 20 years, several postgraduate courses and continuing education programs have been developed to address difficult healthcare conversations. Many of these educational programs have taught specific communication skills protocols and have focused on specific fields, such as oncology [13,14], palliative care [15], internal medicine [16], and general practice [17]. Postgraduate programs typically have been developed for physicians or residents [13,15,18] and enrolled relatively few non-physician healthcare professionals [16]. Overall, there has been a lack of interdisciplinary pedagogical models based on clear theoretical frameworks that can be tailored to teach a broad spectrum of difficult conversations.

To address these needs, in 2002, the Program to Enhance Relational and Communication Skills (PERCS) was developed at Boston Children’s Hospital. The interdisciplinary program is based on a clear theoretical framework [10] and aims to combine the teaching of specific communication skills with the cultivation of relational attitudes. In 2008, the PERCS-Italy program was implemented in Milan, Italy, after one of the co-authors (G.L.) apprenticed in Boston for two years as a Fulbright Scholar to learn the model. Since then, the program has been offered in Italy as a continuing medical education program to interprofessional clinicians to teach about a wide range of difficult conversations. The aim of this study is to describe the experience of the PERCS-Italy program and to assess the participants’ perceived improvements of self-reported competencies.

## 2. Materials and Methods

### 2.1. The PERCS-Italy Program

In 2008, following a period of collaboration between Boston Children’s Hospital and University of Milan, the PERCS program was adapted to the Italian context and implemented at San Paolo Hospital, a public University hospital based in Milan [19]. Preliminary data on the development of the PERCS program at San Paolo Hospital, 10 years after its inception, are reported in a previous contribution in Italian [20]. Since 2016, the PERCS program has also been offered at Humanitas University Hospital, a private hospital based in Milan. In both hospitals, PERCS-Italy has been implemented with the same educational format and length (Table 1). Based on interest and demand, we developed several PERCS-Italy workshops addressing different clinical areas and communicative challenges including PERCS-Emergency Medicine, PERCS-Dialysis, PERCS-Oncology, PERCS-Informed Consent, PERCS-Medical Error, PERCS-Sexuality, PERCS-Organ Donation, PERCS-Vaccination, and PERCS-End of Life.

Each PERCS workshop lasts approximately 5 h and enrolls a maximum of 10–15 interdisciplinary professionals, at all levels of experience. The workshops are led by a team of two facilitators with training in clinical psychology and expertise in healthcare communication and, when possible, with a professional with a medical-nursing background (e.g., nurse, physician). The pedagogical principles of PERCS are creating safety for learning; emphasizing moral and relational dimensions of care; suspending hierarchy among participants; valuing self-reflection; and honoring multiple perspectives [10].

The core educational elements of PERCS workshops include enactments of case scenarios portrayed by participants and actors and subsequent debriefings. The case scenarios are developed with a team of clinical experts in the particular field (e.g., oncologists and oncology nurses for PERCS-Oncology) to assure realistic representative difficult situations for the topic of the workshop. Two of the case scenarios used in PERCS-Medical Error are provided in Table 2 as an example. In the simulations, one or two volunteers participate in their familiar clinical roles, as themselves, in highly realistic simulated enactments. Generally, volunteers are asked to portray their actual disciplinary role in the simulation in order to promote realistic and relevant learning. For this reason, professionals without a clinical role do not typically participate in the simulations but learn from observing, offering feedback, and exchanging perspectives. The participant-volunteers are protected by the simulated learning approach, ensuring a safe learning environment where no patients can be inadvertently harmed. Simulations take place in a separate room and are projected simultaneously to the other participants via an audio-video system. In contrast to the original American PERCS workshops that employs professional actors and includes family faculty members as core components of the programs, in the PERCS-Italy workshops patients and family members are portrayed by clinical psychologists, who have acting training and experience in healthcare communication.

Following each simulation, clinicians and patient-actors who took part in the enactment rejoin the learning group and a collective debriefing is led by the facilitators. The debriefing is conducted according to a learner-centered approach [22]. The discussion generated during the debriefing is therefore co-constructed by volunteering clinicians, actors, participants and facilitators. The debriefing begins by asking the volunteering clinicians what stood out for them and about their experiences, insights, and questions regarding the conversation with the patient or family [10]. The debriefing then unfolds by giving the opportunity to patient-actors [23], other participants, and facilitators to offer constructive feedback. Workshops culminate with the sharing of “take-home messages” by each participant and facilitator in order to summarize and promote the integration of the learning experience.

### 2.2. Participants

PERCS-Italy workshops were advertised through the hospitals’ intranet and newsletter. Participants enrolled voluntarily in PERCS-Italy workshops and attended workshops at San Paolo University Hospital and Humanitas University Hospital between 2008–2020. The PERCS workshop are open to all professionals who work at each of the two hospitals, without any exclusion criteria. However, as the program focuses on difficult healthcare conversations and its attendance contributes to earn continuing medical education credits, clinical professions might be more motivated to participate, rather than staff and administrators.

### 2.3. Measures

To assess the participants’ perceived improvements of self-reported competencies and the adaptability of the PERCS-Italy program in public and private hospital settings, all participants, across both hospital sites, were asked to complete self-reported pre- and post-questionnaires, as developed and incorporated by the American-based authors [24]. The questionnaires were completed immediately before and immediately after the workshop. The pre-questionnaire included sociodemographic data such as gender, professional discipline, and years of experience of the participants.

The self-reported questionnaires included 5-point Likert scale items assessing: (1) sense of preparation; (2) communication skills; (3) relational abilities; (4) self-confidence; and (5) degree of anxiety when engaging in difficult conversations. The post-questionnaire included three additional questions inquiring about the perceived usefulness of the workshop (5-point Likert scale), quality of the workshop (5-point Likert scale), and participant’s recommendation of the workshop to other colleagues (yes/no format).

### 2.4. Data Analysis

Participants’ socio-demographic characteristics and responses to yes/no questions were analyzed through descriptive statistics. Frequencies were also used to describe the participants’ perception of the usefulness and quality of the workshop. *T*-tests for paired samples were conducted to examine the difference in participants’ ratings on preparation, communication and relational skills, confidence, and anxiety before and after the workshops. T-test analyses were also performed on the same dimensions for the two hospitals. Additionally, repeated-measures ANOVA (with Bonferroni post-hoc comparisons) were used to assess differences in participants’ ratings by discipline. For this purpose, based on the different professions and on the frequencies, participants were grouped into physicians, nurses, psychosocial professionals (including psychologists, educators, and social workers), and other professionals (including chaplains, physiotherapists, lab technicians, pharmacists, risk managers, and front-desk officers). We combined all these professionals into the “other” category for two reasons. First, the workers included in the other category are less exposed to a direct communication of difficult news. Second, as the number of each profession is very small, especially for chaplains, pharmacists, risk managers, and administrators, for statistical reasons we decided to aggregate them. Chi square was conducted to assess differences in the program’s ratings of usefulness and quality by discipline. Data were analyzed using SPSS version 26.0 for Windows. Statistical significance was set at *p* < 0.05 for all comparisons.

### 2.5. Ethics

As the study was part of the educational program assessment, it was deemed exempt from the San Paolo Hospital Ethical Committee as per national regulations. All participants signed an informed consent for their data to be published for research purposes.

## 3. Results

### 3.1. Sustainability and Development of PERCS Workshops

**Figure 1:**
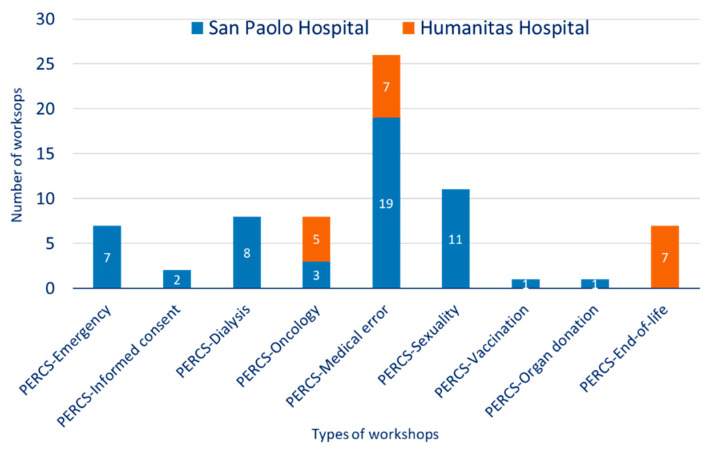
Program to Enhance Relations and Communication Skills-Italy (PERCS-Italy) offerings across two hospitals.

Between 2008–2020, 72 PERCS-Italy workshops were offered (52 at San Paolo Hospital; 20 at Humanitas University Hospital). Across the two settings, different PERCS workshops were offered, based upon the interests of the settings, including PERCS-Emergency Medicine (*n* = 7), PERCS-Dialysis (*n* = 8), PERCS-Oncology (*n* = 9), PERCS-Informed Consent (*n* = 2), PERCS-Medical Error (*n* = 26), PERCS-Sexuality (*n* = 11), PERCS-Organ Donation (*n* = 1), PERCS-Vaccination (*n* = 1), and PERCS-End of Life (*n* = 7). The different PERCS workshops offered in the two hospitals are depicted in Figure 1.

The differences in the workshop offerings are based on the specialties present in the two hospitals. San Paolo Hospital is a general hospital and therefore was interested in a variety of topics. Humanitas Hospital, with a specialty cancer center, was more interested in topics of oncology and end-of-life. Generally, PERCS was initiated because it was congruent with the hospitals’ mission to provide high-quality patient care. Once PERCS was initiated, new workshops were developed based on the current public-health debate (e.g., PERCS-Vaccination), as well as the hospitals’ interests and investments (e.g., PERCS-Organ Donation or PERCS-Medical Error). Some PERCS workshops were terminated because all clinicians were trained, physician leadership co-facilitating the workshop transferred to another hospital, or because interest in the topic had waned.

PERCS has been continuously supported by the two hospitals with public and private funds. In the public hospital, as funding is limited, we offer a limited number of workshops per year and one of the two facilitators conduct the workshops during office hours as a part of her job description and responsibilities. In the private hospital, as the funding is more consistent, we offer more workshops per year. An assistant serves the PERCS program across both hospitals to coordinate email correspondence, material preparation, questionnaire collection, and continuing medical education credits. Facilitators and actors are the same across the two hospitals. In both hospitals, PERCS is offered free of charge to the participants. In 2020, due to COVID-19, live-workshops were suspended. The transition to an online format is ongoing and a new PERCS-COVID is under development.

### 3.2. Participants’ Characteristics

A total of 830 interdisciplinary participants enrolled in the PERCS-Italy workshops, including 297 nurses, 269 physicians, 80 psychosocial professionals, and 171 other professionals (including chaplains, physiotherapists, lab technicians, pharmacists, risk managers, and administrators).

Participants were predominantly female (80.7%), Italian (95.9%), with a mean age of 42 years (SD = 10.5; range: 20–68), and a mean of 16 years of experience (SD = 10.6; range: 0–45). Socio-demographic characteristics of participants of PERCS-Italy are summarized in Table 3, along with the participants’ distribution across the various PERCS workshops.

### 3.3. Perceived Improvements after PERCS Workshops

On pre-post questionnaires, participants reported a significant improvement in preparation, communication skills, relational skills, self-confidence, and anxiety (Table 4). Improvement in all the dimensions was observed in both hospitals, without differences.

Repeated-measures ANOVA showed no differences among participants’ ratings of relational skills (F(3) = 2.27, *p* = 0.079) and anxiety (F(3) = 1.18, *p* = 0.317) by discipline. However, repeated-measures ANOVA revealed significant differences by discipline regarding preparation (F(3) = 3.37, *p* = 0.018), communication skills (F(3) = 5.88, *p* = 0.001), and confidence (F(3) = 4.25, *p* = 0.006). The Bonferroni’s post-hoc tests showed greater improvements for nurses and other professionals as these participants reported lower baseline self-assessment of these skills and this had greater opportunity for improvement. The baseline and post-test mean scores for each discipline are reported in Table 5.

The majority of participants (92.2%) responded that PERCS-Italy workshops were useful or very useful and 84.4% assessed the quality as very good or excellent. Almost all participants (99.2%) would recommend the workshop to other colleagues. No differences were found in the participants’ ratings of usefulness, quality, or recommendation of the program by discipline (Table 6).

## 4. Discussion

PERCS-Italy improved participants’ self-perceived preparation, communication and relational skills, confidence, and anxiety when facing difficult conversations [19]. The findings of PERCS-Italy efficacy do not differ across the two hospitals, thus suggesting its transferability and efficacy across different hospital settings. These data are remarkably consistent with the results of the American-based PERCS [24,25,26,27,28,29], suggesting the cross-cultural veracity and utility of the educational paradigm across multiple healthcare subspecialties.

After the workshops, nurses and other professionals reported a greater improvement in preparation, communication, and confidence compared to physicians and psychosocial professionals. It is possible that nurses and other professionals have had less exposure and opportunity for educational advancement in this area compared to physicians and psychosocial professionals who are typically involved in communication of difficult news and, therefore, may already feel somewhat prepared, confident, and skilled in such conversations. In addition, it is likely that nurses and other professionals, by reporting lower baseline assessment of these skills compared to physicians and psychosocial professional, presented greater room for improvement. Interestingly, no difference was found across disciplines with respect to the improvements in relational attitudes and anxiety. It is possible that these dimensions, traditionally neglected by healthcare training but central in the PERCS program, are particularly amenable to greater learning across professions. PERCS utility, quality, and worthiness of recommendation were recognized by most participants, with no differences across discipline.

It is possible that the self-perceived improvements as a result of the workshops rest with the innovative features of PERCS pedagogy. PERCS pedagogy aims to integrate the teaching of specific knowledge with the promotion of communication and relational abilities, self-reflection, and self-awareness. Adoption of learner-centered facilitation during the workshops, interdisciplinary participation, and use of experiential methodologies encouraged participants to emotionally engage in the learning, appreciate teamwork, and to feel safe so as to step into the learning authentically. Other effective healthcare communication training, in contrast to PERCS, focuses on teaching specific protocols and behaviors. These include Oncotalk [14], a four-day workshop structured around small-group skill practice with simulated patients for medical oncology residents, and Codetalk [16], an experiential communication skills workshop developed for internal medicine trainees and nurse practitioner students with the aim to improve their ability to communicate bad news and express empathy. However, similar to Lipkin’s model of faculty training [11], PERCS embraces the principles of learner-centered (or self-directed) learning and the focus on core human values, such as unconditional positive regard for others and attention to affect [30].

Compared to other postgraduate training programs, which have been implemented in specific clinical settings such as oncology [13] or palliative care [15], the PERCS model has been adapted for interdisciplinary clinicians across a broad array of difficult conversations. PERCS has a standardized approach incorporating simulation-based experiential learning yet is flexible enough to accommodate a range of clinical contexts and types of challenging conversations. Moreover, PERCS is intentionally designed as interdisciplinary to promote appreciation of multiple perspectives, learning from others, and teamwork [12].

Another characteristic feature of the PERCS model is the construction of a learner-centered, respectful experience, which can serve as a model for a patient- and family-centered practice [10]. The connection of PERCS programs within the wider paradigm of patient-centered care might contribute to preserve the balancing between the two most important advantages of PERCS: its codified format and its flexible adaptability to different contexts. Within the panorama of medical education, this connection might highlight the importance for clinicians of tailoring standardized protocols to specific situations and adopting a personalized approach to care.

Importantly, PERCS is not a highly directive or expert-driven model but rather is open and responsive to the participants’ insights, pre-existing knowledge and relational abilities, contributions, and experiences, which co-construct the learning [10]. During the debriefings, PERCS facilitators have objectives for the discussion but these are held in unison with the unique needs of the learning group as they arise from the experiential case scenarios. For these reasons, PERCS debriefings can and sometimes do unfold in unexpected ways, reflecting the unique needs of participants and the patient-actors’ feedback. Facilitators are respectful of the dynamics that occur in the simulations between learners and patient-actors, using these as a springboard to integrate knowledge and sensibilities from the broader literature and field. In this way, PERCS facilitators model for clinicians a flexible and adaptive relational style and help clinicians to develop a relational perspective that considers the point of view of the patient, family members, and other colleagues. By including actors as co-facilitators during debriefings, the patient and family perspectives are prioritized and represented, and the feedback is reported as a trustworthy lived experience, not merely theoretical. A recent study reported that learners identified authenticity, feedback from actors, patient/family perspectives, emotion, and improvisation as key educational elements of PERCS programs [23].

Considering the experience of PERCS-Italy over these 12 years, some critical issues emerge that are related to the evaluation of the program in terms of participants’ actual behavioral change, assessment of long-lasting effects of training, and representativeness of the findings. Lack of resources and the challenge of identifying adequate psychometrically validated measures prevented us from implementing follow-ups and assessing actual behavioral change. The current results of the PERCS-Italy program are based on self-reported perception of improvement and, therefore, reflect perceived attitudinal changes and skills, but not necessarily actual improvements in clinical practice. The effect of the training on participants’ actual behavior has been investigated through follow-up narratives by US colleagues [25,26,31]. Research projects aimed at assessing the influence of PERCS workshops on communication and relational skills, actual interactions with patients, and patient outcomes should be implemented and systematically evaluated [16]. Moreover, there is a lack of data on PERCS-Italy regarding the participants’ self-reported competencies at follow-ups. The study of Meyer et al. [25] and Thiel et al. [31] are two studies assessing the long-term improvements of PERCS training, albeit through narrative self-reports. Finally, there is the issue regarding the external validity of the evaluation findings. PERCS participants, including those who attended the PERCS-Italy workshops, typically enroll on a volunteer basis and, thus, there might be some selection bias in who attends the program and the sample might not be representative of the population. The findings regarding the appreciation and self-reported improvements of the program might have been positively influenced by the motivation and sensibility of the participants who value communication and relational learning relative to difficult conversations.

Efforts to improve the evaluative rigor of PERCS-Italy are needed. These should include measures of actual individual, team, and organizational communicative and behavioral change, as well as the impact on patients’ and families’ perceptions of PERCS training [32]. Future developments of the program might include incorporation of ongoing unit-based educational rounds to consolidate the learning, as has been reported in a sample of over 1000 PERCS learners to support clinicians’ ability to navigate workplace communication challenges while promoting interprofessional teamwork and self-care [33]. To date, the PERCS-Italy has retained the original one-day format, with the possibility for participants to re-attend workshops or to enroll in different PERCS programs, but without opportunities for ongoing or advanced-level training. Initiatives aimed at offering training opportunities after and beyond traditional day-long workshops should be pursued in order to support and consolidate the learning process. To advance our PERCS-Italy program, we could imagine the implementation of ongoing educational, supportive rounds based on actual difficult cases brought by participants aimed to facilitate the transfer of skills into actual practice. Future directions might also include the launching and evaluation of an online PERCS format to promote inclusiveness, reach a greater number of clinicians, and adapt to the social distancing inherent in the COVID-19 pandemic.

## 5. Conclusions

From its first implementation in 2008, the PERCS-Italy program has continuously expanded its educational offerings to be responsive to the various clinical needs of the Italian healthcare context. The study involved a total of 830 clinicians from two hospitals who, after the workshop, reported improved preparation, communication-relational skills, self-confidence and decreased anxiety in dealing with difficult conversations. The data collected during these first twelve years of activity are indicative of the educational relevance and role assumed by the PERCS-Italy program.

## Figures and Tables

**Table 1:** The format of the Program to Enhance Relations and Communication Skills (PERCS)-Italy program.

Strategies	Activities
Warm-up	Pre-test assessment
	Introduction of PERCS principles
	Participants introductions
Brainstorming	Sharing perspectives on “what works in difficult conversations…”
Didactic lecture	Theoretical framework
Active learning	Simulation I with actor
	Group debriefing
	Simulation II with actor
	Group debriefing
Cognitive integration	Take-home points
	Post-test assessment

Adapted from Lamiani et al. [19].

**Table 2:** Case scenarios used in the PERCS-Medical Error workshop.

Case scenario I—Massimo Pecca		Case Scenario II—Lucia Molteni
Massimo Pecca is a 65-year-old man hospitalized for a lung lobectomy due to a tumor of the lung. After surgery, the physician orders an infusion of morphine for pain management at 1.0 mg/h, using the “trailing zero”, a practice prohibited by hospital policy. The nurse, not accustomed to the trailing zero, reads the order as 10 mg/h, not the intended 1 mg/h. The nurse calls the physician to express her concern about the high dose and asks the reason for such a high dose, without specifically mentioning the actual dosage. The physician replies that the dose he ordered (1 mg/h) is appropriate for the intervention. The nurse then starts the infusion at 10 mg/h precipitating a respiratory arrest and transfer to the ICU. After admission in the ICU, Massimo recovers completely. The physician and the nurse meet with the daughter, while Massimo is recovered in the ICU, to communicate the error.		Lucia Molteni is a 41-year-old woman who has been hospitalized for a caesarean section. On the first post-partum day, the physician advises a transfusion of two bags of blood components due to blood loss (around 900 cc) and anemia. The patient agrees to the transfusion but asks that the bags be covered because of an aversion to the blood. The nurse submits the physician’s request to the transfusion center, where only one operator is present. The transfusion center operator, being the only two bags required in the morning, delivers the bags without checking them. The nurse having asked for Lucia Molteni’s bags, takes the bags without checking the name of the request and performs the transfusion by covering the bags as requested. After a while, Lucia begins to feel unwell, trembles, and sweats, and the doctor decides to suspend the infusion. The patient receives 1 gr of flebocortid and 1 mg of a benzodiazepine, with an immediate resolution of the symptoms. Upon discarding the bag, the clinical team realizes they have administered blood to Lucia which is incompatible with blood type. The patient’s change in renal function parameters is so compromised that it requires treatment of two sessions of dialysis. The healthcare team have to communicate to Lucia the error and the new treatment required.

Adapted from Troug et al. [21].

**Table 3:** Participants’ socio-demographic characteristics and distribution across Program to Enhance Relational and Communication Skills (PERCS) editions.

Characteristics	Total Participants (*n* = 830)	San Paolo University Hospital (*n* = 602)	Humanitas University Hospital (*n* = 228)
Discipline, *N* (%) Nurses Physicians Psychosocial professionals ^a^ Other professionals ^b^ Valid N	297 (37) 269 (33) 80 (10) 171 (20) 807 (100)	200 (34) 195 (33) 71 (12) 118 (20) 584 (100)	97 (44) 74 (33) 9 (4) 43 (19) 223 (100)
Years of experience, mean (sd), range	15.8 (10.6), 0–45	16.1 (11), 0–45	15.0 (9.3), 1–40
Age, mean (sd), range	41.6 (10.5), 20–68	42 (10.7), 20–66	40.1 (9.7), 22–68
Gender, *N* (%) Female Valid N	647(81) 802 (100)	458 (79) 580 (100)	189 (85) 222 (100)
Ethnicity, N (%) Italians Valid N	768 (96) 801 (100)	567 (98) 579 (100)	201 (91) 222 (100)
Previous learning experience in communication, N (%) None Coursework Practical experience Residency Continuing education Other Multiple choice of above Valid N	299 (36) 45 (5.4) 165 (19.9) 66 (7.9) 74 (8.9) 56 (6.7) 125 (15.1) 830 (100)	220 (36.6) 29 (4.8) 109 (18.1) 41 (6.8) 46 (7.6) 32 (5.3) 125 (20.8) 602 (100)	79 (34.6) 16 (7) 56 (24.6) 25 (11) 28 (12.3) 24 (10.5) 0 (0) 228 (100)
Mentor/role model, N (%) Yes Valid N	260 (33) 784 (100)	184 (33) 566 (100)	76 (35) 218 (100)
Type of PERCS, N (%) PERCS-Emergency Medicine PERCS-Dialysis PERCS-Oncology PERCS-Informed Consent PERCS-Medical Error PERCS-Sexuality PERCS-Organ Donation PERCS-Vaccination PERCS-End of Life Valid N	86 (11) 84 (10) 99 (12) 24 (3) 309 (37) 127 (15) 16 (2) 10 (1) 75 (9) 830 (100)	86 (14) 84 (14) 37 (6) 24 (4) 218 (36) 127 (21) 16 (3) 10 (2) - 602 (100)	- - 62 (27) - 91 (40) - - - 75 (33) 228 (100)

^a^ This category includes psychologists, educators, and social workers; ^b^ This category includes chaplains, physiotherapists, lab technicians, pharmacists, risk managers, and administrators.

**Table 4:** Participants’ perceived improvements of preparation, communication skills, ability to develop and maintain relationships, confidence, and anxiety after the Program to Enhance Relations and Communication Skills-Italy (PERCS-Italy) workshops.

Dimensions	Baseline Mean (sd)	Post-Test Mean (sd)	Paired-Sample *t*-Test	Cohen’s d	95% Confidence Interval
Preparation Communication Relationship Confidence Anxiety	2.60 (0.93) 2.80 (0.84) 3.14 (0.89) 2.72 (0.88) 3.06 (1.00)	3.33 (0.73) 3.35 (0.73) 3.47 (0.72) 3.37 (0.75) 2.77 (0.85)	−23.74 * −19.22 * −10.72 * −22.49 * 8.84 *	−0.87 −0.71 −0.40 −0.83 0.33	−0.80–−0.68 −0.61–−0.50 −0.38–−0.26 −0.71–−0.60 0.22–0.35

*p < 0.001.

**Table 5:** Participants’ perceived improvements after PERCS-Italy workshops by discipline.

Discipline	Preparation			Communication Skills			Relationship			Confidence			Anxiety		
	Baseline Mean (sd)	Post-Test Mean (sd)	F	Baseline Mean (sd)	Post-Test Mean (sd)	F	Baseline Mean (sd)	Post-Test Mean (sd)	F	Baseline Mean (sd)	Post-Test Mean (sd)	F	Baseline Mean (sd)	Post-Test Mean (sd)	F
			4.23 *			5.89 **			2.27			3.37 *			1.18
Physicians	2.83 (0.95)	3.52 (0.68)		3.10 (0.79)	3.50 (0.68)		3.36 (0.79)	3.57 (0.66)		2.96 (0.88)	3.52 (0.76)		2.93 (0.99)	2.69 (0.85)	
Nurses	2.59 (0.81)	3.22 (0.68)		2.70 (0.78)	3.29 (0.71)		3.02 (0.84)	3.41 (0.68)		2.67 (0.81)	3.32 (0.69)		3.10 (0.97)	2.85 (0.83)	
Psychosocial professionals	2.73 (0.99)	3.51 (0.68)		2.94 (0.90)	3.39 (0.70)		3.26 (0.93)	3.54 (0.71)		2.81 (0.91)	3.39 (0.68)		3.03 (0.98)	2.64 (0.87)	
Others	2.17 (0.96)	3.15 (0.86)		2.37 (0.87)	3.15 (0.83)		2.90 (1.07)	3.31 (0.89)		2.33 (0.91)	3.21 (0.80)		3.19 (1.04)	2.78 (0.91)	
Total	2.60 (0.93)	3.34 (0.73)		2.80 (0.86)	3.34 (0.73)		3.14 (0.90)	3.46 (0.73)		2.72 (0.89)	3.37 (0.74)		3.05 (0.99)	2.76 (0.86)	

* *p* < 0.05; ** *p* < 0.001.

**Table 6:** Participants’ ratings of usefulness, quality, and recommendation of the program by discipline.

Partecipants’ Satisfaction	Physicians	Nurses	Psychosocial Professionals ^a^	Others ^b^	X^2^	*p*
**Usefulness of the program, %** Not at all/little Somewhat Quite/very useful	0.8 11 88.2	0 4.9 95.1	0 4.1 95.9	0.6 6 93.4	18.37	0.244
**Quality of the program, %** Poor/fair Good Very good/excellent	1.6 20 78.4	0.7 11.5 87.8	1.4 17.8 80.8	0.7 10.1 89.2	14.61	0.263
**Recommendation of the program, %** Yes	98.4	99.6	98.6	100	4.85	0.563

^a^ This category includes psychologists, educators, and social workers; ^b^ This category includes chaplains, physiotherapists, lab technicians, pharmacists, risk managers, and administrators.

## Data Availability

The data presented in this study are available on request from the corresponding author. The data are not publicly available due to restrictions regarding the Ethical Committee Institution.
